# Endometriosis: An Immunologist’s Perspective

**DOI:** 10.3390/ijms26115193

**Published:** 2025-05-28

**Authors:** Jenny Valentina Garmendia, Claudia Valentina De Sanctis, Marian Hajdúch, Juan Bautista De Sanctis

**Affiliations:** 1 Institute of Molecular and Translational Medicine, Faculty of Medicine and Dentistry, Palacky University, 779 00 Olomouc, Czech Republicmarian.hajduch@upol.cz (M.H.); 2 Czech Advanced Technologies and Research Institute (CATRIN), Institute of Molecular and Translational Medicine, Palacky University, 779 00 Olomouc, Czech Republic; 3 Laboratory of Experimental Medicine, University Hospital Olomouc, 779 00 Olomouc, Czech Republic

**Keywords:** endometriosis, cytokines, inflammation, autoimmunity, cancer, therapy

## Abstract

Endometriosis, a complex inflammatory disease, affects a significant proportion of women of reproductive age, approximately 10–15%. The disease involves the growth of endometrial glands and stroma outside the uterine cavity, leading to tissue remodeling and fibrosis. Hormonal imbalances, accompanied by local and general inflammation and pain, are key features of endometriosis. Endometriotic lesions are associated with the overproduction of cytokines, metalloproteinases, prostaglandins, reactive oxygen radicals, and extracellular vesicles. Genetic predisposition and cytokine gene polymorphisms have been documented. Macrophages, dendritic cells, mast cells, Th1 in the early phase, Th2 in the late phase, and T regulatory cells play a crucial role in endometriosis. Reduced NK cell function and impaired immune vigilance contribute to endometrial growth. The strong inflammatory condition of the endometrium poses a barrier to the proper implantation of the zygote, contributing to the infertility of these patients. Cytokines from various cell types vary with the severity of the disease. The role of microbiota in endometriosis is still under study. Endometriosis is associated with autoimmunity and ovarian cancer. Hormonal treatments and surgery are commonly used; however, recent interest focuses on anti-inflammatory and immunomodulatory therapies, including cytokine and anti-cytokine antibodies. Modulating the immune response has proven critical; however, more research is needed to optimize treatment for these patients.

## 1. Introduction

Endometriosis is a prevalent chronic inflammatory condition affecting 10 to 15% of women of reproductive age, which incurs substantial healthcare costs [1,2]. This disorder is characterized by the growth of endometrial tissue outside the uterus, resulting in inflammation and fibrosis. The displaced tissue experiences cyclical changes akin to those of normal endometrial tissue. Multiple risk factors contribute to the onset of endometriosis, including familial history of the disease, nulliparity (the condition of never having given birth), early onset of menstruation (menarche), and exposure to various environmental influences. The condition predominantly affects women aged 25 to 45 [3] and is associated with elevated rates of obstetric complications [4,5] and a diminished quality of life [6].

The diagnosis of endometriosis is often delayed, with a typical gap of 7 to 12 years from the onset of symptoms to a surgical diagnosis [7,8]. The condition is systemic, affecting 50–80% of women with pelvic pain, and is a common cause of unexplained infertility [9]. It disrupts the function of the fallopian tubes and interferes with embryo transport, with 25–50% of women undergoing fertility treatments being treated for this condition [9]. Additionally, endometriosis impacts liver and adipose tissue metabolism, which leads to systemic inflammation and altered brain gene expression and contributes to pain sensitization and mood disorders [9].

Chronic inflammation, immune cell phenotype, and function changes are associated with endometriosis [3,5,10]. There are disturbances in neutrophils, monocytes/macrophages, dendritic cells, natural killer (NK) cells, B cells, and T cells [11]. While benign, endometriosis exhibits cancer-like behaviors, including hyperplasia and invasive growth [11,12]. The eutopic endometrium in affected women exhibits molecular abnormalities, which activate oncogenic pathways and increase the production of estrogen, cytokines, prostaglandins, and metalloproteinases, thereby supporting endometrial implant survival [11,12]. Autoimmunity is also observed in patients with endometriosis [13].

In this review, we discuss the immune system’s involvement in endometriosis, the critical role of cytokines, the mechanisms of inflammation and pain, and a brief overview of anti-inflammatory, cytokine, and anti-cytokine treatments.

## 2. Endometriosis

The hypothesis of endometriosis encompasses several mechanisms, including retrograde menstruation, metaplasia, and genetic susceptibility [13,14,15,16,17,18]. The Sampson theory explains that during menstruation, endometrial cells can survive and invade pelvic structures through tubal reflux, leading to ectopic lesions. However, it does not fully explain the mismatch between the high incidence of reflux of menstrual blood (90%) and the lower incidence of endometriosis (10%) [17]. No single theory comprehensively accounts for the various clinical presentations and lesions of endometriosis, including those outside the abdominal cavity or in men [18]. Research has also highlighted proangiogenic factors, such as VEGF, IL-1β, and TNF-α, which play a crucial role in the vascularization of endometriosis [19]. Furthermore, patients with carbohydrate antigen 125 levels (≥35 U/mL) have a higher risk of pelvic adhesions and more extensive lesions [20].

Endometrial tissue growing outside the uterus causes symptoms like chronic pelvic pain, menstrual pain, painful sex, and infertility in 50% of patients [2,11]. The development of endometriosis may involve factors such as retrograde menstruation, immune response issues, and inflammation triggered by adipokines like leptin [21]. Diet and gut microbiota also play a role in influencing symptoms [22]. Endometrial implants depend on estrogen for growth, and there is often an imbalance between estrogens and progestogens, along with progesterone resistance [23]. Inflammation plays an essential role in the pathophysiology of endometriosis. Inflammation is responsible for pain, tissue remodeling, lesion formation, fibrosis, and infertility (decreased ovarian reserve, reduced oocyte quality, impaired endometrial receptivity) and can promote malignant transformation [5,24,25]

There are three types of endometriosis: superficial peritoneal disease (15–50% of patients), ovarian endometrioma (2–10%), and deep infiltrating endometriosis (20%) [26]. Deep endometriosis features nodules deeper than 5 mm and is the most aggressive form, linked to more significant pain and infertility [15,27]. Symptoms may relate to lesion appearance, and treatment response varies by lesion type, with undifferentiated lesions usually being deep infiltrating [28].

Endometriosis and adenomyosis, while benign, exhibit malignant traits like rapid growth and invasiveness. Transitioning from adenomyosis to a premalignant tumor involves genetic and epigenetic changes [29,30]. Integrin β3 (ITGB3) is upregulated in ectopic endometrial stromal cells from endometriosis patients, promoting cell proliferation and invasion [31,32,33]. ITCH, a ubiquitin E3 ligase involved in endometriosis, is downregulated in this condition and, when overexpressed, enhances the ubiquitination of ITGB3, affecting the proliferation and invasion capabilities of ectopic cells [31,32,33]. The opposing expressions of ITCH and ITGB3 suggest that dysregulation of the ubiquitin process may play a crucial role in endometriosis pathogenesis [33]. Additionally, *HOTAIR* lncRNA influences the invasion and migration of endometrial stromal cells via the miR-519b-3p/PRRG4 pathway [12].

Table 1 summarizes the clinical phenotypes associated with endometriosis, highlighting its relationship with pain, infertility, and potential comorbid medical conditions. Diagnosing this condition can be complex due to its similarities with other clinical entities, lack of awareness among healthcare professionals, normalization of symptoms in society, and variability in clinical presentation.

The table shows a difference between autoimmune diseases encountered in patients with endometriosis. The most frequent systemic autoimmune diseases are lupus erythematosus, Sjögren’s syndrome, and rheumatoid arthritis [13]. However, there are also patients with tissue-specific autoimmune diseases, thyroid diseases, Crohn’s disease, and Addison’s disease, which have very different characteristics [11,13]. The connection between endometriosis and autoimmune disease is discussed later in the text.

### 2.1. Genetic and Epigenetic Changes in Endometriosis

Endometriosis has genetic and environmental causes with polygenic inheritance. Relatives of affected individuals are seven times more likely to develop the disease. A twin study indicates that about 52% of disease variance is genetic, and six genetic markers linked to endometriosis have been identified.: CISD2, EFRB, GREB1, IMMT, SULT1E1, and UBE2D3 [35]. Additional analyses have pinpointed associations with loci on chromosomes 7p15.2, 2p25.1 (GREB1), and 12q22 near VEZT [35]. A recent Genome-Wide Association Study (GWAS) and integrative-omics analyses highlight the role of immunopathogenesis and key signaling pathways (Wnt, NOTCH, TGFβ) in regulating endometrial cell behaviors in endometriosis [36].

Endometriosis increases the risk of epithelial ovarian carcinoma, including clear cell, endometrioid, and low-grade serous types [37,38,39]. Carcinogenesis is linked to an imbalance of reactive oxygen species, antioxidants, and systemic inflammation. Endometriotic cysts have high free iron levels, leading to oxidative stress [39]. Abnormal microscopic features of endometriosis, such as atypical cytology and architecture, can be either benign or malignant and have been observed in patients with ovarian cancer [40]. A clear connection between endometriosis, ovarian cancer, and genetic predisposition is observed [41,42,43,44,45,46,47,48]. An integrated analysis of DNA profiles was used to analyze candidate genes for ovarian endometriosis. Lei and coworkers [41] were able to show that the most relevant genes for ovarian endometriosis are TMEM184A, GREM2, SFN, KIR3DX1, HPGD, ESR1, BST2, PIK3CG, and RNASE1. Some of these gene candidates are also associated with cancer: TMEM184A is a prognostic marker in cervical squamous cell carcinoma and endocervical adenocarcinoma [41]; GREM2 inhibits cancer progression and is associated with the inhibition of adipogenesis [42], SFN [41], KIR3DX1 [41], HPGD [43], ESR1 [44], BST2 [45]; PIK3CG [41], and RNASE1 [41].

Mutations in ARID1A, PIK3CA, and PTEN may drive the progression from benign endometriosis to cancer [46,47]. Additionally, these cancers may display mutations in K-RAS and β-catenin/Wnt, along with microsatellite instability, indicating shared genetic susceptibility [48,49]. Exome sequencing revealed that 79% of deep endometriosis patients had somatic mutations; nevertheless, these mutations alone are insufficient for malignant transformation [49].

Chou and coworkers [50], studying the genetics of killer inhibitory receptors (KIRs) in Chinese patients with endometriosis, reported an increase in the number of patients with centromeric A/A haplotypes and a decrease in KIR2DL2, an inhibitory gene of the B haplotype. On the other hand, Marin et al. [51] reported a significant association of KIR2DL2 with the risk of deep endometriosis in Euro-descendants [51]. KIR2DL2 is associated with impaired NK cytotoxic activity and clearance of ectopic endometrial cells [50,51]. A Japanese study found a lower frequency of activating KIR3DS1 and a higher frequency of the inhibitory KIR3DL1+/HLA-Bw4+ combination [52]. It can be concluded that extensive studies are required to define the relationship between KIRs and endometriosis.

The NOD-like receptor (NLR) pathway fundamentally regulates interleukins, proinflammatory cytokines, and NF-κB activity. Single-nucleotide polymorphisms (SNPs) of the NOD1 and PYDC2 genes were associated with endometriosis, whereas SNPs of the NOS2 and PYDC1 genes were not [53]. Other studies have identified additional cytokine gene polymorphisms associated with the disease, including IL1A rs2856836 and rs2856836 [54]; rs11575812, rs2069772, and rs2069762 [55]; IL-10 (rs1800872) and IFN-γ a13 allele [56]; IL-12B rs3212227 [57,58], IL-16 rs11556218, rs4778889 [59], and rs4778889 [60]; IL-17A rs2275913 [61]; IL-18 SNP rs1946518 [62]; association with severity (TNF, rs1800629); IL-1beta (IL1B, rs1143634) and IL1-Ra (rs2234663) [63]; and macrophage migration inhibitory factor (MIF) rs755622 [64]. IL-8 SNP rs4073 has been related to pelvic pain in endometriosis [65]. These reports show a clear association with disease and disease severity. However, most studies focused on only one target cytokine, and integrative studies are required.

The epigenetic changes associated with endometriosis include DNA methylation and phosphorylation, modifications to histones and non-coding RNA, and chromatin remodeling and organization [66]. Specific epigenetic abnormalities have been described in endometriosis that alter the expression of key transcription factors. For example, hypomethylation of the GATA-binding factor-6, accompanied by overexpression, transforms an endometrial stromal cell into an endometriotic phenotype [66]. Steroidogenic factor-1 overexpression causes excessive estrogen production, which drives inflammation via pathologically high levels of estrogen receptor-β [67].

Some miRNAs serve as biomarkers for endometriosis and could be targets for therapy [68,69]. Some of these miRNAs are shared between endometriosis and atherosclerosis, both diseases are linked [70]. The most relevant miRNA-detected changes in plasma and serum are (1) increased miRNA 122, 199a, 125 b-5p, 150-5p, 342-3p, and 451a, and (2) decreased miRNA Let-7b, Let-7d, Let-7f, 17-5p, 20c, 20a-5p, and 3613-5p [67,68,69,70,71]. Studies on non-coding and circular RNA and endometriosis are ongoing [72].

Impaired endometrial decidualization reduces fertility in endometriosis. Transcriptomic profiling shows alterations in pathways, including defective BMP/SMAD4 signaling, oxidative stress response, and retinoic acid signaling [70]. Constitutive NF-κB activation in endometriotic lesions promotes inflammation, invasion, and angiogenesis while inhibiting apoptosis [73,74,75]. Active endometriosis lesions become fibrous, resulting in the adherence of tissues and organs [76].

High levels of BCL-6 (a transcription factor) in women with endometriosis are associated with decreased activation of progesterone receptors, resulting in progesterone resistance in the endometrium [77]. The BCL6 gene is significantly upregulated in ectopic tissues compared to tissue from healthy controls [78]. mRNA levels of estrogen-related receptors β and γ (ERRβ and ERRγ) were substantially lower in ectopic tissues from patients with severe endometriosis than in the eutopic endometrium of healthy controls [79].

The activation of mutated K-RAS in donor endometrial epithelium and stroma promotes lesion growth in a murine model of endometriosis but is insufficient for cancer transformation [80]. Essential factors for the progression from endometriosis to endometriosis-associated ovarian cancer include somatic mutations in ARID1A, K-RAS, PTEN, and microsatellite instability [81,82]. Overall, there is a link between genetic predisposition and polymorphism for endometriosis, along with other factors under investigation: signal transduction modulation, miRNA, long coding, and circular RNA.

### 2.2. Extracellular Vesicles

Extracellular vesicles (EVs) are membrane-bound particles that transport regulatory molecules like proteins, miRNAs, and lipids. They consist of small EVs (sEVs), such as exosomes, and large EVs (lEVs), also known as macrovesicles, which are released from various cellular compartments [83]. Gram-positive and Gram-negative bacteria can generate apoptotic bodies and extracellular vesicles (BEVs) [84]. BEVs can be formed from the microbiota in the endometrial fluid and can induce the secretion of TNF, IL-6, and IL-17, which are involved in endometriosis [84].

Vesicles of different sizes are found in follicular fluid and affect follicle size, oocyte function, promote granulosa cell proliferation, and cell survival under stress [83,85]. Small and large EVs differ in number, morphology, specific membrane markers, and miRNAs [83,84]. Large EVs influence steroidogenesis by affecting enzyme mRNA levels, stimulating estradiol secretion via the PI3K/AKT pathway [83,85]. Newly identified mitochondria-derived EVs that contain mitochondrial proteins have a potential role in fertilization [83].

Nazri et al. [86] reported the isolation of exosomes from peritoneal fluid. The concentration varied by cycle phase and disease stage. Proteomic analysis revealed specific proteins in exosomes from endometriosis patients that were absent in healthy controls. Five proteins found exclusively in the endometriosis groups are PRDX1, H2A type 2-C, ANXA2, ITIH4, and tubulin α-chain [87]. Moreover, tissue-derived exosomes downregulated NKG2D-mediated cytotoxicity by containing NKG2D ligands MICA/B and ULBP1-3 and the proapoptotic molecules FasL and TRAIL [87]. The presence of these ligands impairs the immune response against endometrial tissue.

Patients with endometriosis exhibited a higher percentage of particles testing positive for platelet biomarkers than the total number of EVs [88,89]. Platelets create a procoagulative state in endometriosis patients and transport miRNA, including miRNA15b-5p and 65 [90]. These findings suggest a potential role for platelets in the development of endometriosis. They are found in lesions, contribute to fibrosis in damaged tissue [75,88,89], and are associated with extracellular vesicles [91]. Nevertheless, the impact of platelets on endometriosis still requires more research.

### 2.3. Microbiota and Endometriosis

Microbiota plays a role in establishing and progressing endometriosis [92,93]. The gut microbiota may influence estrogen production and local immune inflammation, promoting endometrial cell proliferation [92,93,94,95,96]. Estrogen metabolism entails a comprehensive three-phase process that includes hepatic conjugation, microbial deconjugation, and subsequent excretion [93,94]. Within the liver, estrogen undergoes conjugation to form water-soluble metabolites, such as estrone sulfate and estradiol glucuronide, which facilitate biliary excretion into the gastrointestinal tract [94]. The gut microbiota plays a crucial role in this process, particularly through specific bacteria such as *Clostridium*, *Escherichia*, *Bacteroides*, and *Lactobacillus*, which produce the enzyme β-glucuronidase [94]. This enzyme deconjugates estrogen metabolites, permitting their reabsorption into the systemic circulation. This phenomenon, called enterohepatic recirculation, is essential for regulating estrogen bioavailability and maintaining hormonal homeostasis [94,95,96]. Dysbiosis, characterized by an imbalance in gut microbial composition, can significantly disrupt estrogen metabolism [94]. Reducing β-glucuronidase-producing microbes may hinder estrogen reabsorption, potentially resulting in systemic estrogen deficiency, adversely affecting reproductive and metabolic functions [94,95,96]. Conversely, an overabundance of these bacteria may lead to excessive estrogen recirculation, which has been linked to estrogen-dependent conditions such as breast cancer, endometriosis, and infertility [94,95,96].

The endometrial environment and peritoneal cavity microbiota have been linked to endometriosis [97,98,99,100,101,102]. Increased levels of *Gardnerella*, *Streptococcus*, *Escherichia*, *Shigella*, and *Ureaplasma* were noted in the cervical microbiota of endometriosis patients [98]. Distinct microbial communities were found in feces and peritoneal fluid, with increased pathogens in peritoneal fluid and reduced protective microbes in feces [88,89]. Endometriosis patients exhibited lower alpha and beta diversity in gut microbiota compared to controls, with significant differences in the abundance of several bacterial classes [101]. The Firmicutes/Bacteroidetes ratio, indicative of dysbiosis, was also higher in endometriosis patients, alongside notable differences in various taxa [101]. The relationship between endometritis and endometriosis has been documented [102,103,104]. Clinical trials targeting dysbiosis and endometrial lesions could benefit cases of recurrent implantation failure and pregnancy loss [104].

Modulating gut microbiota could potentially slow endometriosis progression. Sobstyl et al. [105] noted that interactions among microbiota and dysbiosis may activate immune cells, producing proinflammatory cytokines that disrupt stem cell homeostasis and affect estrogen levels. Certain gut bacteria, like *Bacteroides* and *Lactobacillus*, secrete enzymes that elevate free estrogen levels [106]. An increase in *Escherichia coli* has been observed in the feces of endometriosis patients, but the interactions between gut, vaginal, and endometrial microbiota remain unclear [104,107].

Patients with chronic pain and endometriosis had lower alpha diversity than controls, showing increased levels of vaginal *Streptococcus anginosus* and rectal *Ruminococcus* [106,107]. Guo et al. [108] speculated that different Gram-negative bacteria, such as *Escherichia coli*, residing in the vagina could be involved in the pathogenesis of endometriosis in humans. In addition, gut microbiota promotes the progression of endometriosis by influencing peritoneal immune cell populations. Then, the onset and development of endometriosis may be related to the abnormal immune response caused by gut dysbiosis [108].

## 3. Immune Response in Endometriosis

### 3.1. Pattern-Recognition Receptors (PRRs), Pathogen-Associated Molecular Patterns (PAMPs), Damage-Associated Molecular Patterns (DAMPs), and Endometriosis

PRRs can be classified into five families: Toll-like receptors (TLRs), C-type lectin receptors (CLRs), NOD-like receptors (NLRs), retinoic acid-inducible gene I-like receptors (RLRs), and AIM2-like receptors (ALRs) [109,110,111]. Their activation leads to proinflammatory cytokine, interferon production, phagocytosis, and cell death [109,110,111]. PAMPs include lipopolysaccharides, flagellin, viral RNA, and fungal cell walls [109,110,111]. DAMPs are various molecules, such as proteins (e.g., amyloid beta, HSP70), metabolites (e.g., ATP, uric acid), ions (Ca^2+^, K^+^), and nucleic acids (self RNA, DNA) [112].

Endometriosis may develop in two distinct phases. The initial wave occurs with an infection and TLR activation. The second wave is characterized by sterile inflammation resulting from oxidative stress and receptor activation by DAMPs [111,112,113].

Increased TLR2 B cells and myeloid dendritic cells correlate with severe endometriosis [111,112,113,114]. Individuals with endometriosis have significantly higher TLR2 and TLR9 concentrations in peritoneal fluid than healthy controls [115]. In a mouse model, *Ureoplasma urealyticum* infection promotes endometriosis by enhancing inflammatory mediators and MMP-2 expression via TLR2 signaling [115]. Additionally, ectopic endometriotic lesions show heightened TLR3 and TLR4 mRNA expression compared to eutopic tissues [116,117,118].

Inflammasomes are multi-protein complexes, particularly the NLRP3 inflammasome, which activate inflammatory caspases [119]. NLRP3 binds to procaspase-1, activating caspase-1, which cleaves pro–IL–1β and pro–IL–18 into their active forms [119,120]. This mechanism has been linked to endometriosis’s pathogenesis, with increased caspase-1, IL-18, and NLRP3 expression observed in ectopic endometrial tissue [121,122]. Granulosa cells from women with endometriosis show elevated levels of the NLRP3 inflammasome and increased IL-1β and IL-18 in follicular fluid, contributing to infertility [123,124]. NLRP3 expression is significantly higher in ovarian endometriosis, and using an NLRP3 inhibitor has effectively reduced ovarian endometriosis lesions in animal models [125,126].

Interactions between macrophages and endometrial stromal cells via NLRP3 signaling enhance stromal cell migration and endometriosis progression [127]. NLRP3-deficient mice had smaller endometrial lesions, but this was reversed with wild-type macrophages [127]. Ectopic endometrial tissues showed elevated IL-18, IL-6, and IL-1β mRNA levels compared to eutopic endometrium and controls [128,129]. NLRP3-mediated pyroptosis is associated with fibrosis via TGF-β1, and inhibiting it may reduce fibrosis in endometriosis [124,130]. TRIM24 potentially facilitates endometriosis progression through the NLRP3/caspase-1/IL-1β pathway [131,132]. High estrogen receptor β levels in endometriotic lesions correlate with increased IL-1β, promoting cell adhesion and proliferation [132]. Progesterone inhibits NLRP3 activation in normal stromal cells via autophagy, but this effect is reduced in endometriotic cells [133].

NLR family CARD domain-containing 5 (NLRC5) acts as a negative regulator in endometriosis by inhibiting inflammation [134,135]. Its overexpression increases autophagy in ectopic endometrial stromal cells, while inhibition decreases it [135]. NLRC5 levels are higher in the ectopic and eutopic endometria of endometriosis patients compared to those with leiomyoma, peaking in the ectopic endometrium, and it suppresses IL-6 and TNF-α [136]. This suggests that NLRC5 overexpression inhibits estrogen receptor β-mediated development and inflammatory responses in endometriosis [134,135,136].

C-type lectin receptors (CLRs) play a key role in the innate immune system by recognizing carbohydrates [137]. In patients with endometriosis, peritoneal fluid exhibited increased CLR MR2 and DAP12 mRNAs, alongside decreased galectin levels [137,138,139]. The mannose receptor C, type 2 (MRC2), was found to be lower in ectopic endometrial stromal cells compared to normal ones, whereas peritoneal dendritic cells in endometriosis showed heightened mannose receptor expression [139]. Additionally, the receptor for advanced glycation end products (RAGEs) is associated with endometriosis and infertility [140], with soluble RAGEs (sRAGEs) potentially impacting in vitro fertilization success [140]. The functions of RAGEs and CLRs continue to be explored.

### 3.2. Innate Immune Response in Endometriosis

Table 2 and Table 3 provide a general overview of endometriosis’s innate and adaptive immune involvement. The aim is to give the reader a summary of the most critical issues in endometriosis.

The innate immune cell response comprises several protein elements, with the complement pathway and defensins being the most relevant. The complement system, a component of innate immunity, contains over 50 proteins that aid in eliminating pathogens, removing immune complexes and apoptotic debris, and participating in processes such as inflammation, adaptive immunity, coagulation, metabolism, tissue regeneration, and host–microbiota symbiosis [141,142]. Table 2 shows several pathway components that have been related to endometriosis. On the other hand, defensins produced by Paneth cells, neutrophils, and epithelial cells have not been involved in endometriosis (Table 2).

Macrophages are crucial in endometriosis physiopathology. Chronic macrophage stimulation and high iron levels in the peritoneal cavity elevate reactive oxygen species in women with endometriosis [143,144,145]. Estrogen prompts peritoneal macrophages to secrete cytokines and prostaglandins through estrogen receptor-β, which decreases MMP-9 activity and inhibits phagocytosis [25]. Upon cell activation, NF-κB p65 phosphorylation induces the transcription of proinflammatory cytokines (TNF-α, IL-1β, IL-6, IL-8), proangiogenic factors VEGF, growth factors like FGF-2, and adhesion molecules [146,147], and COX-1 and 2. COX-2 is responsible for the increased concentrations of PGE2 in the peritoneal fluid [148]. The co-culture of macrophages with endometrial stromal cells enhances the proliferation and invasiveness of these endometrial stromal cells [149].

Proinflammatory peritoneal fluid in women with endometriosis elevates FasL expression in regurgitated endometrial cells, enhancing Fas-mediated cell death of activated immune cells and aiding immune evasion by endometrial cells [150,151]. Macrophages initiate a regenerative program vital for lesion growth. In patients with endometriosis, peritoneal macrophages exhibit higher iron storage than controls [146,152] and have difficulty managing elevated hemoglobin levels in the peritoneal fluid [153,154,155,156]. In women with endometriosis, peritoneal macrophages show heightened proinflammatory markers of the M1 phenotype, while M2 macrophages often shift toward M1 [154,155,156]. The presence of two subpopulations of macrophages in the lesion was reported using single-cell analysis [156] and the role of M2a in fibrogenesis [157]. In the advanced stages of the disease, there is an increase in M2 macrophages and a decrease in the M1 type; the opposite occurs in the initial stages (I–II) [145,158]. Macrophages activated by IL4 can induce epithelial-to-mesenchymal transition and fibroblast-to-myofibroblast transdifferentiation through the production of TGF-β1 [145,152].

Uterine NK (uNK) cells express CD56 but no other classical NK cell or T cell markers. The number of uNK cells changes during the menstrual cycle, pregnancy, and various endometrial pathologies [159]. There is an increase in uNK in the mid-secretory phase [151]. CD56+ cells remain high during early pregnancy and comprise 70% of the lymphocytes at the interface between maternal decidua and the invading trophoblast [159]. Approximately 10% of uNK are CD56+ CD16+, while 90% of the population has the CD56+ CD16− phenotype [159]. In the peripheral blood, 90% of NK cells are CD56+ CD16+ (pNK); CD56+ CD16+ and CD56+ CD16− uNK cells exhibit functional differences. CD16+ cells are cytolytic, whereas CD16− uNK cells secrete cytokines [159]. Activated uterine natural killer (uNK) cells regulate trophoblast invasion into the decidua [159]. The elevation of CD56+ cells is higher in infertile women and pregnancy loss and appears to be directly correlated with pelvic endometriosis [160].

Limited information exists on the roles of neutrophils and eosinophils in the endometrium [161] since they are not commonly observed in the endometrium or vagina except for infectious diseases. Nonetheless, mast cells are therapeutic targets for treating endometriosis, inflammation, infertility, and pain. A recent review [162] describes the different experimental treatments involving mast cells in animal models.

Myeloid-derived suppressor cells (MDSCs) are a diverse group of immature myeloid cells, including dendritic cells, granulocytes, and monocyte/macrophage precursors, known for their immunosuppressive properties [163]. They play a significant role in the progression of immunological disorders, such as chronic inflammation and cancer. MDSCs can be categorized into two primary types: polymorphonuclear (PMN) MDSCs, also referred to as granulocyte (G) MDSCs, and monocytic (M) MDSCs. The significant outcome of MDSC expansion is immunosuppression, which may lead to angiogenesis and the secretion of cytokines or growth factors, potentially exacerbating the progression of conditions such as endometriosis [163,164,165,166].

While the proportion of PMN-MDSCs in both peripheral blood and peritoneal fluid was significantly higher in patients with endometriosis, the proportion of M-MDSCs did not differ between control subjects and those with endometriosis [166,167]. On the contrary, an abnormal expansion of M-MDSCs in peripheral blood and peritoneal fluid of patients with endometriosis [167]. Additionally, MDSCs are more abundant in ectopic endometrium than in normal endometrium [168]. M-MDSCs, alongside inflammatory cytokines and exosome miRNA, appear to be involved in the progression of endometriosis [169]. Cysteine–Cysteine Chemokine Receptor 5 (CCR5) and its ligand, CCL5, could drive the progression of endometriosis by increasing the accumulation of MDSC [169]. On the other hand, MDSCs drive the process of endometriosis by enhancing angiogenesis [170].

Another critical issue in endometriosis is the role of immature dendritic cells in the lesions. Those cells are inefficient in antigen presentation and are inducers of tolerogenic responses [171]. The lack of mature dendritic cells in endometriosis is also related to the increase in Tregs, and as stated before, the effectiveness of the Tregs depends on the milieu. Li and coworkers [172] have postulated using dendritic cells for therapeutic use in endometriosis. The effectiveness of this proposal can likely be assessed soon.

**Table 2:** Innate immune proteins and cells in endometriosis.

Component	Characteristics	Ref
Complement pathway	Increased expression of the protein components of the pathway in human endometriosis.	[173]
	Increased levels of C1q, C1 inhibitor, mannose-binding lectin (MBL), C3c, C4, and the membrane attack complex (SC5b-9) in the peritoneal fluid of endometriosis patients. Increased expression of 1QA, C1QB, C1R, C1S, C2, C3, C4A/B, C5, C6, C7, C8A, CFB, CFH, and CFI in ectopic endometrium.	[174]
	C1q levels are correlated with vessel formation in endometriosis (human).	[175]
	The lectin pathway may not be involved in endometriosis in humans.	[176]
Defensins	There are no changes in defensin levels in women with endometriosis.	[177]
Neutrophils	Increased levels of human neutrophil peptides 1, 2, and 3 have been observed in the endometrial fluid of women with endometriosis.	[178]
	In endometriosis, neutrophil phagocytosis is impaired. Neutrophils support the survival of endometrial cells and help create a microenvironment conducive to the development and growth of lesions (mouse and human).	[179,180,181]
	Neutrophil depletion in mice reduces the formation of endometriotic lesions.	[181]
Macrophages	Elevated IL-8, C-C chemokine RANTES (CCL5), MCP-1, and MIF attracted more cells in advanced endometriosis lesions in humans and mice.	[182]
	There are different types of macrophages present in endometriomas (mouse model).	[183]
	High iron levels in the peritoneal fluid impair the phagocytic response and increase oxygen radical formation (human).	[173,179,184]
	Extracellular vesicles modulate macrophage response in endometriosis.	[185,186]
	The expression of CD36 in macrophages is inhibited by the high concentration of PGE2 in the endometrioma (human).	[187]
	An increased expression of CD200 correlates with reduced phagocytic activity and decreased CD36 expression in endometriosis (human).	[188]
	TLR4 and RAGE expression in peritoneal fluid macrophages inversely correlate with endometriosis severity (human).	[189]
	Macrophages play a vital role in both fibrosis and mesenchymal transdifferentiation (in humans and mice).	[190,191]
NK cells	There is a higher density of CD56 in uNK cells in patients with endometriosis undergoing IVF treatment.	[159,160,161]
	Uterine NK cell amounts are higher in patients with endometriosis.	[159,161,192]
	There is a decrease in tissue immature CD56 cells following the surgical removal of endometriomas (human).	[159,193]
	NK cell cytotoxic activity is significantly reduced in women with moderate to severe endometriosis (peripheral, peritoneal, and uNK).	[194,195]
	Granzyme B and perforin secretion were reduced in NK cells from endometriosis patients.	[195,196]
	The increase in soluble MICA/B levels in the peritoneal fluid of patients with endometriosis negatively affects the cytotoxic function of NK cells.	[196]
	Elevated levels of IL-6 and TGF-β1 in the peritoneal fluid of endometriosis patients are responsible for the impaired cytotoxic activity of NK cells.	[197,198]
	High IL-15 levels produced by ectopic endometrial stromal cells can inhibit NK cell function (human).	[199]
	IL-10 produced from co-cultures of macrophages can also inhibit NK cell cytotoxic response (mouse and human in vitro).	[200]
	NK cells from patients with endometriosis have a high density of NK inhibitory receptors and ligands. However, NK-activating receptors are also expressed at high levels.	[201,202,203]
Mast cells	High numbers of degranulated mast cells have been found in women with endometriotic lesions.	[204,205]
	Increased concentrations of stem cell factor in the peritoneal fluid of women with endometriosis are responsible for increased mast cell migration.	[162]
	Mast cells express estrogen receptors and are highly activated by the estrogens in the ectopic endometrium in patients with endometriosis.	[205]
	Mast cells are involved in pain in women with endometriosis.	[206]
Dendritic cells	Increased immature cells (CD80lowCD1ahigh) and fewer mature cells (CD80highCD1alow) in the peritoneal fluid (mouse and human).	[207,208]
	The activity of enzyme 1-hydroxysteroid dehydrogenase type 1, which activates cortisol, impairs dendritic cell maturation in patients with endometriosis.	[209]
	CD1c expression on peripheral myeloid dendritic cells was higher during menstruation in patients with endometriosis.	[210]
	IL-10 produced by dendritic cells induces angiogenesis in patients with endometriosis	[211]

### 3.3. Adaptive Immune Response

Table 3 highlights the role of T and B lymphocytes in the adaptive response in endometriosis. Changes in CD8 populations are essential, while CD4 cell modifications can be categorized into early Th1 and late Th2 responses. Inflammatory lesions promote the production of anti-inflammatory cytokines to balance the inflammatory environment, revealing a complex local cytokine storm beyond just immune cell mediators.

In a recent review by Knez et al. [212], it becomes clear that different Tregs subpopulations, resting Tregs (rTregs; Foxp3loCD45RA+ T cells), suppressive Tregs (Foxp3hiCD45RA−), and non-suppressive Tregs (non-Tregs; Foxp3loCD45RA− T cells), should be considered when analyzing Tregs in endometriosis [212]. Tregs expressing CTLA-4 induce tolerogenic responses (reduced T cell activation and proliferation), while IL-17 and TGFβ are crucial for lesion formation and fibrosis, respectively. Tregs interact with T follicular cells, B cells, dendritic cells, and macrophages [212]. The suppressive response involves the induction of M2 macrophages and the production of IL-1, thereby decreasing the inflammatory milieu.

The role of B cells in endometriosis is less clear than that of T cells [213]. The generation of anti-endometrial autoantibodies [214] and the production of IL-17 and, in certain conditions, IL-35, illustrate the complex nature of cell interaction [11,213]. More research is required to understand the role of these cells in endometriosis and their probable link with autoimmune diseases.

**Table 3:** Adaptive immune cells in endometriosis.

Cell Type	Characteristics	Ref
T cells	In patients with endometriosis, circulating CD8+ cells and activated T cells increase, leading to higher secretion of proinflammatory cytokines and elevated autoantibody titers.	[214,215,216]
	CD8 cell apoptosis is elevated in endometriosis patients due to Fas-FasL interaction.	[151]
	Foxp3+CD39+CD73+ Treg cells are decreased in the blood of women with deep infiltrating endometriosis but increased in the peritoneum and endometriotic lesions.	[217,218]
	Elevated levels of estrogen and thymus-expressed chemokine (TECK/CCL25) lead to an increase in Tregs, which in turn reduces immune surveillance in endometriosis patients.	[219]
	Disruption of Th17/Treg balance leads to heightened inflammation in ectopic and eutopic endometria in women with endometriosis.	[220,221]
	Peritoneal fluid from endometriosis patients promoted Treg cell generation and inhibited Th17 cell differentiation in CD4+ T cell cultures in vitro.	[221]
	Patients with endometriosis have higher amount of CD16+ CD8 T cells in their peripheral blood, and CD8 T cell cytotoxicity is increased in menstrual effluent.	[222]
	Patients with endometriosis show low amounts of perforin-CD8 T cells in peripheral blood.	[223]
	Potential T cell exhaustion indicated by PD-1 expression and increased PD-1L presence in tissues of endometriosis patients.	[224]
	The Th2 immune response (IL-4, IL-10) dominates later stages of endometriosis, whereas Th1 is present initially. CTLA-4 plays a role in chronic inflammation and endometriosis in humans and mice.	[178,225,226,227]
	Higher soluble circulating CTLA-4 levels in patients with endometriosis are associated with chronic inflammation.	[227]
	Estrogen plays a role in regulating the GATA3 transcription factor and Th2 differentiation in patients with endometriosis.	[228]
	The interleukin IL-4/IFN-γ, IL-10/IFN-γ, and IL-4/IL-2 ratios are higher in women with endometriosis, probably in the late stage.	[229]
B cells	Increased circulating levels of activated B cells in patients with endometriosis.	[230,231]
	Local B cells secrete high levels of IL-6 and IL-17, inducing local inflammation. They also produce anti-endometrial antibodies.	[231]
	The production of IL-35 by B cells is increased in patients with endometriosis.	[232]

## 4. Cytokines and Endometriosis

Cytokines play a critical role in generating endometriosis since the inflammatory milieu in endometriosis leads to poor quality of oocytes and infertility [6]. However, most of the focus of the published studies has referred to cytokines produced by immune cells, which does not represent the whole picture of events in endometriosis. The local tissue production of cytokines must be considered, as well as the role of adipokines, which may also have a dual role, regional and peripheral [22]. A clear example is the role of leptin in regulating the amount of stored energy by binding to specific neurons in the brain.

Alarmins are key inducers of cytokine release by activating DAMP receptors. HMGB1, a byproduct of cell death, enhances proinflammatory cytokine secretion, particularly under hypoxic conditions, making HIF-1α modulation crucial in endometriosis [233,234,235,236]. HMGB1 is also affected by mediators like prostaglandins [237], while the role of leukotrienes depends on LPS induction in the endometrium [237]. Early animal studies showed reduced endometrial inflammation with leukotriene receptor antagonists [237,238,239,240], but the results were inconclusive for human clinical trials.

Lipoxin A4 suppresses inflammation and activates autophagy, which helps reduce the proliferative effects of endometriosis [241,242,243]. Resolvins, as noted in research by Dimitrieva et al. [242] and Gu et al. [243], also contribute to the management of endometriosis by decreasing the inflammatory response through the signal transduction pathways induced upon receptor/ligand interaction [244]. Additionally, resolvins may offer a promising approach to alleviating the pain related to endometriosis [245].

Despite the enormous efforts in analyzing different types of biomarkers in endometriosis reviewed by Collie and coworkers [246], there is no clear consensus on most metabolites. The authors only reported 3-hydroxybutyrate, lactate, phosphatidic acids, succinate, pyruvate, tetradecenoyl carnitine, hypoxanthine, and xanthine as the most consistent biomarkers. Since these intermediate metabolites can be affected by different pathways, more research is required to determine the metabolic pathways involved. Hypoxia should be carefully analyzed as proposed by Wilson [247].

Ferroptosis represents a significant cellular event in endometriosis [248]. Iron within the endometrioma influences the generation of radical species in conjunction with immune cells that provoke proinflammatory and cytokine responses. As highlighted in tumor immunology [249], exploring ferroptosis, radical production, cellular senescence, cell death, and immune exhaustion in endometriosis is paramount. Further research is necessary to identify appropriate pharmacological targets.

Table 4 presents a comprehensive overview of the critical cytokines involved in endometriosis. Depending on the tissue environment, it categorizes these cytokines into proinflammatory, anti-inflammatory, and those with pro- or anti-inflammatory properties. Additionally, the table includes cytokines associated with angiogenesis, cell growth, chemokines, and inhibitory factors. While other mediators may play a role in the physiopathology of endometriosis, they have not yet been thoroughly studied. In addition, some critical issues must be considered: (1) there are differences in sample analysis, serum, peritoneal, or endometrial fluid, and endometrioma analysis; (2) in addition, some analyses were performed in patients with different stages of endometriosis. The table also includes whether the results are from the human, animal model, or in vitro, which is essential, considering possible differences that can be encountered.

The table is divided into three parts: (1) proinflammatory cytokines, (2) anti-inflammatory cytokines, and (3) mixed effects. The separation facilitates the analysis based on the role of the cytokines. The cytokines with mixed effects refer to cytokines whose general description can be anti-inflammatory; however, their role in the lesion may differ.

Table 5 represents the list of adipokines that have been studied in endometriosis. However, it is essential to note that obesity is not prevalent in patients with endometriosis. Patients with endometriosis usually have a low BMI, and obesity increases its severity [298,303,326,327,328]. Various hypotheses have been proposed regarding the potential role of adipokines in endometriosis [326,328]. Even though there are disagreements about the relationship between obesity, BMI, and endometriosis, most researchers support the proposal of a dual effect of adipokines in the tissue and the central nervous system. The finding of adipokines in the lesion and their possible role in lesion growth and modulation of the immune response requires more research.

It is important to note that the precise mechanisms underlying the diverse cytokines involved in endometriosis remain unclear. A comprehensive understanding of the chronological progression of this condition is critical for developing novel treatment strategies aimed at reducing both the growth of lesions and associated pain.

## 5. Mechanisms of Pain in Endometriosis

Endometriosis-associated pain stems from various mechanisms, including nociception, inflammation, and altered pain processing in the nervous system. It is frequently linked to psychological distress and fatigue. Additionally, angiogenesis leads to the growth of nerve fibers that contribute to this pain [341,342]. The size of the lesions appears to be related to pain intensity in patients with lesions on the intestinal wall [342,343,344]. However, there is no significant correlation between the graded severity of morphological characteristics and the intensity and character of pain symptoms [342,343,344,345]. It can be concluded that there is no consistent correlation between endometriosis and reported pain severity.

Two main descriptions of pain occur in endometriosis: (1) Nociceptive pain occurs due to physical damage to non-neural tissues, particularly from endometrial lesions and the surrounding structures, such as the pelvic lining. This type of pain can be classified as visceral, which relates to internal organs, or somatic, which pertains to muscles and skin [346]. (2) Nociplastic pain occurs when the nervous system becomes oversensitive, amplifying pain signals. Nociceptive stimuli can trigger it and persist even after the initial injury has healed. The effect is due to increased sensory nerve density and a reduced density of sympathetic nerve fibers in endometriotic lesions [347].

An imbalance in sensory and sympathetic nerve fiber density within lesions is associated with pain severity in women with endometriosis [346,347,348]. Compared to women without endometriosis, there is an increase in sensory nerve fibers and a decrease in sympathetic nerve fibers, which may contribute to pain [346,347,348]. In women diagnosed with endometriosis, there is a significant elevation in the density of nerve fibers within endometriotic lesions and the adjacent myometrium compared to normal peritoneal tissue. This heightened density, particularly of sensory nerve fibers, positively correlates with the severity of pain patients report [346,347,348]. The potential mechanisms contributing to the severity of pain include (1) sensitization of nociceptors within the endometriotic microenvironment; (2) neurogenic inflammation accompanied by the release of proinflammatory neuromediators; and (3) central sensitization, which involves an amplification of central pain signal processing [342,343,346,347,348].

The interaction between macrophages and nerves constitutes a significant factor in pain associated with endometriosis [347,348,349]. Within this interaction, cytokines are linked to the phenomenon of inflammatory pain [346,347]. Moreover, various immune cells and cytokines can also play a role in the pain observed in lesions [348].

Endocannabinoids and phytocannabinoids possess anti-inflammatory, anti-nociceptive, and anti-proliferative properties that may aid in managing endometriosis, characterized by inflammation, increased vascularity, and pain [350,351,352]. While endometriotic lesions show varying levels of endocannabinoids, their exact role in disease progression and potential bystander effects remains unclear [350,351,352]. In vivo murine model studies indicate that synthetic cannabinoids and specific endocannabinoids, such as palmitoylethanolamide (PEA), possess anti-inflammatory properties and can inhibit the proliferation of endometriosis-like lesions [351]. However, the exact mechanism is still elusive.

According to Farooqi and colleagues [352], both the endocannabinoid system (ECS) and gut microbiota play significant roles in the pathophysiology of endometriosis. The ECS is essential for regulating inflammation and modulating pain perception, while gut microbiota significantly influences immune responses and hormonal equilibrium [352]. Worsening symptoms of endometriosis have been associated with an imbalance in the ECS and gut microbiota, linked to elevated levels of endocannabinoids resulting from alterations in CB1 receptor expression [352]. Furthermore, an increase in *Prevotella* and *Escherichia coli* prevalence within the gut microbiota correlates with exacerbated gastrointestinal and endometriosis symptoms [352]. These dysbioses are also associated with heightened circulating levels of proinflammatory cytokines, such as TNF-α and IL-6 [352]. Nevertheless, elevated endocannabinoids, particularly 2-AG, may confer protective effects on the gut by mitigating inflammation and enhancing gut permeability.

Increased levels of the neurotransmitters glutamate and glutamine were found in the anterior insula of endometriosis patients, enhancing connectivity to the prefrontal cortex (where pain-related memories are stored) [353,354]. Other areas of the brain are also affected. According to Eippert et al. [355], the periaqueductal gray, which is involved in pain-modulatory pathways, is enlarged in individuals with pain, and measurable changes are observed in the thalamus, insula, and putamen [342,353,354,355].

In endometrial lesions, macrophages and nerve fibers interact to promote pain [349,353,354,355,356]. Ectopic endometrial lesions secrete nerve fibers that produce CSF-1 and CCL2, which attract macrophages to the periphery of nerves and regulate their polarization toward the M2 phenotype [356]. On the other hand, macrophages, incubated with CSF-1 and estrogen, produce brain-derived neurotrophic factor (BDNF) and neurotrophin-3 (NT-3), which stimulate neurite growth from ganglia explants [356].

Endometriosis-related pain is classified as neuropathic or neuroinflammatory [357]. Ectopic endometriotic lesions promote inflammation and disrupt the transmission of inflammatory mediators, altering how nerve fibers process and transmit information [357,358]. Disorders that are characterized by sensory dysfunction, such as overactive bladder syndrome and irritable bowel syndrome, are commonly co-diagnosed with endometriosis [357,358]. These comorbidities suggest a more complex pathophysiology for pain in this condition that cannot be explained by endometrial lesions alone [342,343,344,353,354,355,356,357,358]. Chronic remodeling of the nervous system may occur in shared sensory neural pathways to induce a state of protracted peripheral and central sensitization and chronic pain in patients with endometriosis [357]. Microgliosis, astrogliosis, and enhanced substance P neurokinin-1 receptor immunoreactivity have been observed within the spine in mice with endometriosis, suggesting the development of neuroinflammation and the sensitization of spinal circuitry in this condition [359]. Prostaglandin E2, TNFα, NGF, RANTES, IL-8, and IL-1β are elevated within the peritoneal fluid of endometriosis patients [360]. These mediators can all activate sensory nerve endings directly [12,357,358,359,360].

Neuroangiogenesis in ectopic endometriotic lesions explains the transmission of the pain. An increased density of miniature, unmyelinated nerve fibers (sensory afferents, sympathetic, and parasympathetic efferents) has been found in endometrial lesions [360,361]. The local production of VEGF and NGF by macrophages supports neuroangiogenesis [12,360,361,362,363,364]. On the other hand, the activation of sensory afferent nerves initiates the recruitment of mast cells and the subsequent release of proinflammatory cytokines (TNF-α, NGF, PGE2, IL-1β), which contributes to a chronic state of neurogenic inflammation [365]. Neurotrophic factors produced by macrophages, such as Netrin-1, insulin-like growth factor-1, and ten-eleven translocation 3 (TET3), play a role in the pain associated with endometriosis [365,366,367].

Recently, the role of IL-33 in macrophage/neuron-induced pain has been studied [368]. IL-33 enhanced the release of TNF-α and IL-1β, facilitating macrophage recruitment and neurogenesis in ectopic lesions [368]. IL-33 increased the expression of the transient receptor potential vanilloid 1 (TRPV1), which is responsible for the phenomenon [368]. In women with endometriosis and severe chronic pelvic pain, serum IL-16 levels were higher compared to women with mild pain [61].

Tregs may influence endometriosis pain by modulating macrophages to create a local tolerogenic response, which reduces proinflammatory cytokines, decreases cell migration, and mitigates estrogen’s effects on endometriomas [213]. It has recently been found that the meningeal Treg (mTreg) inhibits nociception in female mice [369]. mTreg cells produced enkephalin, which acted on delta opioid receptors in MrgprD+ sensory neurons to reduce pain [369]. However, enkephalin was unnecessary for Treg cell-mediated immunosuppression, and the process depends on sex hormones [369]. One can envision that the understanding of pain in patients with endometriosis is just beginning.

## 6. Endometriosis and Autoimmunity

There is an association between endometriosis and autoimmune diseases. Women with endometriosis may have a higher risk of conditions such as systemic lupus erythematosus, Sjögren’s syndrome, rheumatoid arthritis, celiac disease, multiple sclerosis, and inflammatory bowel disease compared to those without endometriosis [13,370,371,372]. Endometriosis shares similarities with autoimmune diseases, featuring elevated cytokines, B cell activation, T and B cell function abnormalities, autoantibody formation, and decreased apoptosis [13]. Women with endometriosis have alterations in B cell activity and an increased incidence of autoantibodies [13]. These autoantibodies can be directed against various phospholipids, histones, and polynucleotides [13], and against the ovary, endometrium, nucleus, smooth muscle, cardiolipins, sperm, laminin, and lupus anticoagulant [13]. Antinuclear antibodies (ANAs) in pelvic endometriosis appear to be an immunological secondary effect and do not represent an aggravating factor in patients with pelvic endometriosis [373]. A correlation between the diameter of endometriomas and anti-thyroid peroxidase antibodies has been reported [374]. Patients with endometriosis exhibit elevated transferrin and alpha-2-HS glycoprotein levels in their serum and peritoneal fluid, which may contribute to observed autoimmunity to these proteins [375]. However, no recent reports of these autoantibodies (transferrin and alpha-2-HS glycoprotein) and their impact on the disease exist.

Dotan and coworkers [376] have addressed the issue of SARS-CoV-2 and molecular mimicry in endometriosis. Several other triggers of this process may be observed with viral or bacterial infection and local or gut microbiota. This topic opens an interesting point to address from the pharmacological point of view, preventing autoimmunity.

IgG and complement deposits have been found in the eutopic endometrium in women with endometriosis, corresponding to a decrease in the total serum complement levels [175]. This may have been caused by the ectopic endometrium acting as a foreign trigger that induced an autoimmune response, resulting in infertility [175]. It is not yet clear whether the formation of autoantibodies in endometriosis is a natural response to chronic local tissue destruction or a pathological reaction leading to more generalized autoimmune dysfunction [175].

A singular report exists regarding the formation of autoantibodies to GM-CSF in individuals diagnosed with deep endometriosis [325]. Nonetheless, it is highly plausible that autoantibodies against additional cytokines are present in patients with endometriosis, considering the unique characteristics of this population. These autoantibodies could significantly impact the equilibrium of cytokines within the microenvironment and may contribute to the diminished immune responsiveness often observed in endometriosis. Further investigation into this subject is essential, as it may facilitate the classification of these patients while also providing new therapeutic targets and guidelines for treatment.

## 7. Immunological Therapies in Endometriosis

**Figure 1:**
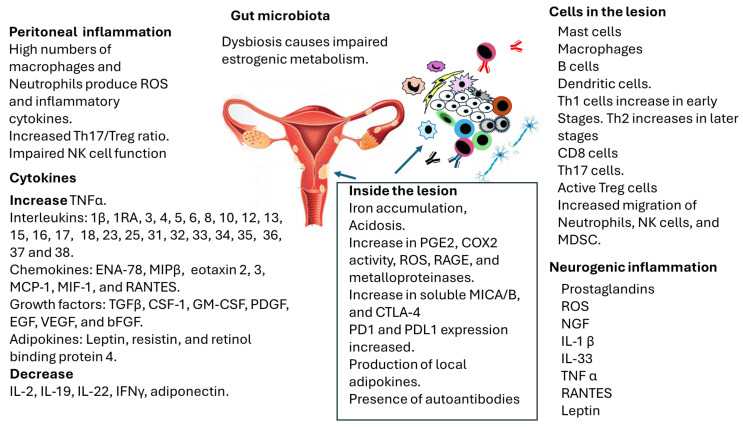
A general summary of endometriosis as discussed in this review.

Figure 1 provides an overview of the cells and processes involved in endometriosis to understand the complexity of the endometrial lesion. Multiple factors generate autoimmunity based on cell death; however, the possible induction of malignancy, mainly ovarian carcinoma, is also represented.

Endometriosis is typically managed through various therapeutic options, including progestogens, combined oral contraceptives, gonadotropin-releasing hormone antagonists and agonists, androgens, aromatase inhibitors, selective progesterone receptor modulators, selective estrogen receptor modulators, nonsteroidal anti-inflammatory drugs, and/or surgical excision of endometriotic lesions [220,377]. However, many of these treatment modalities are associated with adverse effects, particularly concerning the degree of hypoestrogenism, and there is a notable risk of recurrence following the cessation of therapy. Considering the significant inflammatory component of endometriosis, recent research has increasingly concentrated on utilizing anti-inflammatory and immunomodulatory therapies. Table 6 summarizes therapies for endometriosis with immunomodulatory and anti-inflammatory effects, ranging from common drug treatments to natural products. On the other hand, Table 7 is focused on cytokine inhibition or addition.

In a recent review, Zhang et al. [378] examined potential therapies targeting immune-associated factors in endometriosis. The objective is to improve the function of NK cells and macrophages. NK cells can be modulated by blocking inhibitory receptors, using cytokines such as IL-2 and IL-12, or through immune checkpoint therapy (anti-PD-1 or anti-PDL-1) [378]. Currently, this type of therapy has not been tested in patients with endometriosis [378]. Regarding macrophages, potential therapeutic targets include the suppression of the M2 phenotype or the activation of the M1 phenotype. These two approaches are lacking in the treatment of endometriosis. Another possible therapy option may be using C3 inhibitors or the blockade of C5a and C3a [378]. Anti-IL-33 antibody treatment of the endometriosis mouse model slightly, but not significantly, reduced peritoneal inflammation and reduced peritoneal cell concentration compared to the isotype control [379].

Combining immunophilin suppressors with steroid hormones, such as progesterone, may be a promising approach to treating chronic inflammation associated with endometriosis. Tacrolimus, cyclosporine, progesterone, and analogs can effectively suppress FKBP51, a common target of these agents [380].

Three different drugs that can block ribosome biogenesis, including inhibitors against mTOR/PI3K (GSK2126458) and RNA polymerase I (CX5461 and BMH21), were used in a mouse model with human endometriosis features [380]. The lesion numbers were reduced in treated mice compared to those treated with the vehicle [380].

Other treatment options include drugs with antiangiogenic effects, such as those targeting VEGF (anti-VEGF antibody) or inhibiting tyrosine kinase (Sorafenib, Sunitinib, Pazopanib) [19]. All the studies with these agents are in animal models. Another drug with antiangiogenic effects through VEGF receptor-2 is cabergoline (a dopamine agonist). In a clinical trial, this drug reduced endometrioma size more effectively than an LHRH agonist [381]. In another small trial, cabergoline decreased pain in patients with endometriosis [382].

Novel therapeutics have been proposed for the management of endometriosis. Iron chelators have demonstrated promising outcomes in animal models [383,384]. Other strategies aim to ameliorate hypoxic conditions. Sitagliptin has been shown to mitigate hypoxia-induced injury by inhibiting the overproduction of COX-2, PGE2, TNF-α, and IL-6, yielding successful results in animal studies [385,386]. The anti-hypoxic agent myo-inositol trispyrophosphate (IPP) enhances oxygen release from hemoglobin and has effectively inhibited the proliferation of endometrial cells in hypoxic conditions in preclinical models [387]. Research has examined the anti-cancer polypeptide vaccine RESAN, which has been shown to reduce lesion size in mouse models [388]. There are no human reports on the efficacy of this vaccine due to the absence of clinical trials. The use of extracellular vesicles to treat endometriosis presents a promising avenue, particularly for the delivery of miRNA [389]; nevertheless, this research domain necessitates further exploration and the establishment of consensus among the scientific community.

An interesting proposal has been published that involves modulating kisspeptin neurons, impacting the hypothalamic–pituitary axis and controlling LH and FSH, and consequently, endometrial growth [390]. Since the modulation of pain may involve sex hormones and the production of endorphins and sensory neurons [391], it would be interesting to analyze the induction of endorphins as modulators of endometriosis growth in preclinical models. The topic is engaging, and many patients will benefit from therapy. Future research in this area is anticipated to focus on these patients.

Although pharmacological interventions targeting cytokines have not undergone analysis in clinical trials, epidemiological data concerning the efficacy of various inhibitors of TNFα and IL-1β concerning endometriosis and fertility must be examined. Such an examination may unveil novel pathways for both research and therapeutic intervention.

**Table 6:** Therapies for endometriosis with immunomodulatory and anti-inflammatory effects. Traditional drugs and natural products.

Drug	Effects	References
Glucocorticoids	Inhibit the inflammatory milieu in endometriosis. Prevent the self-renewal, migration, and differentiation of endometrial stem cells and endometriosis formation.	[392,393,394]
Statins	Statins reduce inflammation and inhibit the formation of new blood vessels, acting as anti-angiogenic agents in the murine model.	[395,396]
	In a pilot study, administering atorvastatin 10 mg daily for 7 days improved nitric oxide-mediated, endothelial-dependent cutaneous microvascular function in women with endometriosis.	[397]
Pentoxifylline	It reduces inflammation by regulating the immune response.	[398]
	There was an increased tendency for pregnancy after surgery in the group treated with pentoxifylline compared to the placebo.	[399]
	Patients who received pentoxifylline showed significantly improved visual analog scale scores after 3 months. There is insufficient evidence to recommend pentoxifylline for the treatment of subfertility and pain related to endometriosis.	[400,401,402]
Peroxisome proliferator receptor γ (PPARγ) activators	The compounds inhibit cell proliferation, induce apoptosis in endometriotic epithelial and stromal cells, reduce vascularization, and repress VEGF, IL-6, IL-8, and TNF-α gene expression.	[403]
	Ciglitazone decreased the size of ectopic endometriotic tissues in a rat model of endometriosis.	[404]
	In a baboon model of endometriosis, Rosiglitazone decreased the size of the endometriotic lesion. Pioglitazone improved embryo implantation rates in infertile women with endometriosis undergoing IVF by reducing serum RANTES.	[405]
	No clinical trial has been published.	[406]
Rapamycin (mTOR inhibitor)	Rapamycin treatment reduced the volume of lesions in a mouse model of endometriosis.	[407]
	In women with infertility due to endometriosis, rapamycin has improved rates of fertilization, implantation, clinical pregnancy, and live births. More clinical trials are needed to ascertain the possible benefit of rapamycin treatment.	[408]
Bentamapimod (c-Jun N-terminal kinase inhibitor)	In a mouse and rat model of endometriosis, bentamapimod led to a reduction in lesion size.	[409]
	In baboons with induced endometriosis, bentamapimod decreased the lesions’ area and volume. No clinical trial has been published.	[410]
Ligustrazine (Tetramethylpyrazine)	A natural product has demonstrated a broad anti-inflammatory effect in preclinical trials. No clinical trial has been published.	[411]
Resveratrol	Several preclinical trials have published the anti-neoplastic, anti-inflammatory, anti-oxidative, anti-microbial, anti-atherogenic, and anti-angiogenic effects of resveratrol.	[412]
	Prevents the progression of experimental endometriosis in living organisms and reduces the invasiveness of endometrial stromal cells in laboratory tests.	[413]
	Resveratrol reduced MMP-2 and MMP-9 levels in the endometrium and blood of women with endometriosis.	[414]
	Treatment with resveratrol reduced TNF-α2 and VEGF expression in patients with endometriosis.	[415]
	There is not enough evidence to support the use of resveratrol in humans.	[416]
Astaxanthin (antioxidant)	Treatment with astaxanthin reduced serum levels of malondialdehyde, IL-1β, and TNF-α, decreasing IL-6 and TNF-α levels in follicular fluid in one triple-blind placebo-controlled clinical trial of patients undergoing assisted reproduction.	[417]
Curcumin	In ectopic endometrial stromal cells cultured in vitro, it suppresses the TNF-α-induced secretion of IL-6, IL-8, and MCP-1, and the mRNA expression of ICAM-1 and VCAM-1.	[418]
	In eutopic endometrial stromal cells of patients with endometriosis, in vitro treatment inhibits the secretion of IL-6, IL-8, G-CSF, MCP-1, and RANTES.	[419]
	In a small trial involving nano-micellar curcumin, inflammatory and oxidative patterns linked to IVF treatment in patients with endometriosis showed improvement.	[420]
	There is not enough evidence to support the use of curcumin in humans. Well-designed clinical trials are needed.	[421]
Quercetin	Experimental data on quercetin have demonstrated its antioxidant, anti-inflammatory, and anti-angiogenic properties.	[422]
	It decreased the volume of endometriosis lesions in a mouse model. No clinical trials have been published.	[423]
Epigallocatechin gallate (EGCG)	EGCG notably decreased the proliferation, migration, and invasion of endometrial and endometriotic stromal cells in vitro model of human endometriosis. In mouse models, it also reduced the growth of endometrial lesions. No clinical trials with the purified compound have been published, although trials utilizing green tea have shown some improvement.	[424,425]
N-palmitoyl ethanolamine plus trans-polydatin	It induces anti-inflammatory effects in women with endometriosis. It reduced pelvic pain in women after laparoscopy.	[426]
	A meta-analysis showed no conclusive evidence.	[427]
Cannabidiol	It reduced the diameter, volume, and area of lesions in rat models of endometriosis. It exhibited an anti-fibrotic effect, lowering IL-1β, TNF-α, and PGE2 levels in peritoneal fluids.	[428]
	It alleviated pelvic pain and related symptoms. Long-term use may be linked to cannabis use disorder, psychosis, and mood disturbances. No clinical trials have been published.	[429,430]
Fenretinide (synthetic retinoid)	Fenretinide reduces the levels of retinol fatty acid binding protein 4. It is used in cancer and cystic fibrosis, but there are no clinical trials in patients with endometriosis.	[431,432,433,434]
Vitamin D	The effects of vitamin D supplementation have produced controversial results that require further studies.	[435,436,437]

**Table 7:** Cytokine-related treatment for endometriosis.

Treatment	Effects	References
Antibody-based (anti-fibronectin F8) pharmacological delivery of interleukin 4 (F8-IL4)	In a mouse model of endometriosis, F8-IL4 reduced the number and volume of lesions while lowering the expression of genes related to cell adhesion, invasion, and neovascularization, such as integrin β1, MMP-3, MMP-9, and VEGF, without affecting inflammatory cytokines. No clinical studies have been performed in humans.	[438]
IL-12	IL-12 enhances cytokine production and increases NK cell activity. An intraperitoneal injection of IL-12 reduced lesion size in a mouse model by activating NK cells and inhibiting the development of endometriotic lesions. No studies have been performed in humans.	[439,440]
Interferon (IFN) I	In a rat model of endometriosis, the subcutaneous administration of IFN-α reduced the volume of endometriosis lesions.	[441]
	IFN-β1a inhibited the in vitro growth and movement of endometrial stromal cells obtained from patients.	[442]
	IFNα2b treatment increased the later recurrence of endometriosis in a small clinical trial.	[443]
IL-37	Anti-inflammatory effects. In mouse models, IL-37 reduced the size and weight of endometriotic-like lesions and the expression of IL-1β, IL-6, IL-10, TNF-α, VEGF, and ICAM-1 in a murine model of endometriosis. No studies have been performed in humans.	[444,445]
Anti-TNF-α	In patients with endometriomas who were treated using assisted reproductive technology, etanercept was shown to increase the pregnancy rate and double the live birth rate. However, this result was not statistically significant. (*p* = 0.052).	[446]
	In a retrospective study, peri-implantation treatment with TNF-α inhibitor increased the implantation rate and clinical pregnancy rate significantly compared with non-treated controls; however, no changes in the pregnancy rate of live birth were observed. Cochrane reviews of humans with endometriosis did not find conclusive evidence.	[447]
	Epidemiological data on young women treated with anti-TNFα therapy and endometriosis incidence have not been published.	[448]
IL-1 antagonist (anakinra)	In a pilot study using anakinra, mild improvements were observed. A reduction in the inflammatory markers BDNF, IL-1RA, and IL-6 was reported.	[449]

## 8. Conclusions

Endometriosis is an inflammatory disorder characterized by elements of autoimmunity and a reduced state of immune surveillance. This condition is defined by the abnormal proliferation of functional endometrial glands and stroma located outside the uterine cavity, often resulting in significant pain and infertility. The pathogenesis of endometriosis is multifaceted, involving immunological, hormonal, and genetic factors. Cytokines, adipokines, and growth factors are integral components in this process. Furthermore, the ectopic endometrium may display functional properties that differ from the eutopic endometrium. A notable association has been established between endometriosis and ovarian cancer. Autoimmunity is frequently observed in patients diagnosed with endometriosis, and the generation of autoantibodies may be influenced by events occurring within the lesions. Increased iron accumulation, elevated formation of oxygen radicals, and infections (resulting from dysbiotic events within the microbiota) can enhance antigen secretion. Future investigations into molecular mimicry may elucidate the mechanisms underlying the generation of autoimmunity. While anti-inflammatory therapy presents a promising strategy for managing this condition, further clinical studies involving human subjects are necessary to validate its efficacy.

Further epidemiological studies are necessary to investigate the relationship between autoimmunity and endometriosis and to examine the use of immunomodulators among young women to assess the incidence of endometriosis. Additionally, the implementation of cytokine and anti-cytokine therapies in fertility clinics addressing issues such as implantation failure and recurrent miscarriages may yield valuable insights for longitudinal studies and facilitate the development of novel pharmacological treatments for endometriosis.

## Figures and Tables

**Table 1:** Spectrum of the clinical phenotypes in endometriosis based on the literature [2,3,4,9,10,11,13,15,16,34].

Lesion	Clinical Characteristics	Possible Coexisting Medical Conditions
**Pelvic** Superficial peritoneal [9,13] Focal (adenomyoma) or diffuse (adenomyosis) lesions within the myometrium [9,13] Ovarian endometriosis [9,13] Deep endometriosis Lesions > 5 mm [13,15] Common in the rectovaginal septum It may involve the large intestine, bladder, ureters, and appendix [13,15]	**Pelvic pain** Dysmenorrhea [9,13] Dysuria [9,13] Dyschezia [9,13] Dyspareunia [13]	**Pelvic** Cystitis/painful bladder [9,13] Irritable bowel syndrome [13] Anal bleeding [13] Chronic endometritis [9,13] Inflammatory bowel disease [9,10,13] Polycystic ovary syndrome [9,10,13] Ovarian cancer [9,13] Uterine Fibroids (leiomyomata) [9,13] Vulvodynia [13] Possible link with recurrent vaginal infections [9,13]
**Extra pelvic** Thoracic [9,13,15] Diaphragm, lungs, pleura, pericardium Liver and spleen [9,13,15] Abdominal wall [9,13,15] Lymph nodes [9,10,13] Brain [9,13,15] Kidney [9,13,15]	**Infertility** Implantation failure [2,4,9,13] Spontaneous miscarriage [4,9,13] Alterations of tubal structures [4,9,13] Diminished ovarian reserve [9,13] Placenta previa [9] Premature delivery [9]	**Extra pelvic** Fatigue [2,4,9,13] Fibromyalgia [13] Joint disorders [13] Migraine [2,9,13] Systemic [2,4,9,13] Systemic autoimmune diseases (Lupus, Sjögren’s syndrome, Rheumatoid arthritis) [2,13,16,34] Tissue-specific autoimmune diseases (Thyroiditis, Crohn’s disease, Addison’s disease) [2,13,16,34], Immune-related conditions (allergies, chronic inflammation) Thyroid disorders [13,16,34] Mental health conditions (depression, anxiety) [2,9,13]

**Table 4:** Cytokines involved in endometriosis.

Cytokine	Role in Endometriosis	Reference
Proinflammatory cytokines		
IL-1β	Increased levels in the follicular fluid of endometriosis patients.	[26,250,251]
IL-1 RA	Increased levels in the follicular fluid of endometriosis patients. It binds active IL-1β, reducing receptor binding and biological activity.	[252,253]
IL-2	Decreased levels in endometriotic lesions. Increase in soluble CD25 in peritoneal fluid.	[254,255,256,257]
	Increased levels were reported in patients with severe endometriosis.	[258]
IL-3	Increased levels were reported in endometriotic lesions.	[259,260,261]
IL-5	Increased levels were reported in the follicular fluid of endometriosis patients.	[260,261]
IL-6	Increased levels were reported in the follicular fluid of endometriosis patients. It is a proinflammatory cytokine.	[252,253,261]
IL-7	Higher in the eutopic as compared to the ectopic tissue of endometriosis patients.	[262]
	Associated with IL-15 in maintaining endometriosis.	[263]
IL-8	Increased levels were reported in the follicular fluid of endometriosis patients.	[264,265,266]
IL-12p40	Increased levels were reported in the follicular fluid of endometriosis patients.	[267,268]
IL-15	Higher levels were reported in the peritoneal fluid of women with endometriosis.	[263,269,270]
IL-16	Increased levels were reported in the peritoneal fluid of patients with advanced stage endometriosis (III/IV).	[271,272]
IL-17	Elevated levels of IL-17 have been observed in patients during the early stages of the disease. IL-17 promotes the proliferation, invasion, and implantation of endometriotic cells. Additionally, increased IL-17 levels have been linked to higher levels of IL-8, VEGF, CSF-1, and GM-CSF.	[273,274,275]
IL-18	Increased levels have been reported in the peritoneal fluid of endometriosis patients. Affects endometrial receptivity.	[276]
IL-23	Increased levels are observed in the follicular fluid of endometriosis patients, stages III–IV > stages I–II. Involved in IL-17 production and inflammation.	[277]
IL-25	Increased levels were reported in the peritoneal fluid of patients with endometriosis.	[278]
IL-31	Increased levels were reported in the plasma of endometriosis patients	[279]
IL-32	Increased concentrations of IL-32 were reported in the peritoneal fluid of patients with endometriosis. An increase in IL-32 is correlated with elevated levels of IL-8 and CCL2 and enhanced cell proliferation.	[280,281]
IL-34	Increased serum levels in patients with endometriosis. Autocrine production promotes endometriosis.	[282]
IL-35	Enhanced levels are reported in ectopic endometrium. Suppresses immune response, increasing the growth of endometrial cells.	[283]
IL-36α, β, γ and Receptor	Increased levels are reported in the peritoneal fluid of endometriosis patients.	[284]
IFNγ	Decreased levels are reported in the peritoneal fluid of endometriosis patients. It induces macrophage activation (M1) and enhances the proinflammatory response.	[178,256,257]
TNFα	High levels of TNFα are found in patients with endometriosis, particularly at their highest in those with severe endometriosis.	[285,286]
CXCL chemokines	In women with endometriosis or polycystic ovary syndrome, the levels of CXCL1-8, 10, 12, 13, and 16 are increased, while CXCL9 and 14 are decreased.	[287]
FRACTAL-KINE	Decreased levels were reported in the peritoneal fluid of patients with endometriosis.	[288]
	Increased levels were reported in patients with endometriosis.	[289]
MCP-1 (CCL2)	Increased follicular fluid levels in endometriosis correlate with severity and attract neutrophils, NK cells, and lymphocytes linked to RANTES and IL-8.	[290,291]
	Association with hepatocyte growth factor and insulin-like growth factor-1.	[292]
MCP-2/3/4	Increased levels are reported in patients with endometriosis.	[286]
MIP-1α	A decrease in cytokine levels was reported in patients with endometriosis. Increased levels were reported.	[289]
MIP-1β	Increased levels were reported in patients with endometriosis.	[289]
eotaxin 2/3	Increased levels were reported in patients with endometriosis.	[289]
ENA-78	Increased levels were reported in patients with endometriosis.	[289]
RANTES (CCL5)	Increased levels were reported in patients with endometriosis.	[286,289]
MIF-1	Increased levels were reported in patients with endometriosis. The levels are associated with 17β-estradiol. MIF-1 is a proinflammatory cytokine.	[293,294,295]
CSF-1	Increased levels were reported in patients with severe endometriosis.	[296,297,298]
PDGF	Increased levels were reported in the peritoneal fluid of patients with endometriosis.	[298,299,300]
VEGF	Increased levels were reported in the peritoneal fluid of patients with endometriosis. Involved in increased vascularization.	[298,299,300,301,302]
bFGF	Increased levels were reported in the peritoneal fluid of patients with endometriosis.	[298,299,300,301,303]
Anti-inflammatory cytokines		
TGFβ	Patients with severe endometriosis exhibited increased levels of TGFβ, which play a role in the fibrosis observed in these individuals.	[77,304,305]
IL-10	Increased levels were found in the follicular fluid of patients with endometriosis, produced by various cells.	[266,306]
IL-19	A decrease in serum levels of IL-19 has been observed in patients with endometriosis.	[307]
IL-22	A decrease in serum levels was reported in patients with endometriosis.	[308]
	IL-22 is implicated in endometrial cell invasion in humans and mice and carcinoma cell proliferation.	[309,310]
IL-37	Increased levels are reported in the peritoneal fluid of endometriosis patients. Involved in anti-inflammatory response in vitro and animal models.	[253,284,311]
IL-38	Increased levels are reported in the peritoneal fluid of endometriosis patients. Involved in anti-inflammatory response.	[284]
Mixed effects proinflammatory and antinflammatory		
IL-4	Increased levels in the follicular fluid of endometriosis patients.	[312,313,314,315,316]
IL-13	Differential expression in ectopic and eutopic endometrium in endometriosis patients. High levels of the cytokine have been associated with infertility.	[314,317]
IL-27	IL-2 + IL-27 are involved in the growth of human endometrial cells in vitro. Its role in endometriosis is still controversial.	[318]
IL-33	Increased serum levels were reported in the peritoneal fluid of patients with deep endometriosis, which could induce an anti-inflammatory response.	[319,320]
	It is involved in epithelial–mesenchymal transition.	[321,322]
EGF	Increased levels were reported in the peritoneal fluid of endometriosis patients involved in endometrial invasion.	[298,299,300,301,303]
GM-CSF	Increased levels were reported in patients with severe endometriosis. It is controversial since it may have local anti-inflammatory effects.	[29,323,324]
	Autoantibodies against GM-CSF are present in the serum of patients with deep endometriosis.	[325]

**Table 5:** Adipokines involved in endometriosis.

Adipokine	Characteristics	References
Leptin	Elevated leptin levels have been observed in serum and peritoneal fluid in patients with endometriosis.	[327,329,330]
	Researchers found a positive association between leptin levels and endometriosis in the mouse model.	[21,331]
	Controversial results have been reported in humans.	[332]
	Elevated local leptin levels in endometriosis lesions are associated with increased transcription factor HIF-1α.	[333]
	Endometriosis may be related to dysfunctional adipose tissue, which affects metabolism, browning, body weight regulation, and pain pathways.	[21,334]
Adiponectin	Low circulating adiponectin levels in women are associated with endometriosis.	[335,336]
Resistin	Increased concentrations have been reported in women with endometriosis.	[337,338]
	Research suggests a potential correlation between resistin and IL-23 levels.	[277]
Retinol binding protein 4 (RTB4)	Increased plasma levels of RBP4 have been reported in patients with endometriosis.	[339]
	RTB4 may play a role in the infiltration of immune cells in human endometriosis.	[340]
Visfatin/NAMPT and resistin	The three adipokines may be secreted locally within the human endometrioma as part of an inflammatory response, regardless of the stage of endometriosis.	[341]
Ghrelin, GLP-1, visfatin, GLP-1.	A reduction in ghrelin, GLP-1, glucagon, and visfatin levels in the peritoneal fluid of women with endometriosis may contribute to lesion development by proinflammatory macrophages.	[341]

## Data Availability

Not applicable.
